# Insights on Novel Dental Resin Composites: A Scoping Review

**DOI:** 10.3390/polym18202516

**Published:** 2026-10-14

**Authors:** Lau Kian Ping, Matheel AL-Rawas, Eyas Abuhijleh, Sohaib Fadhil Mohammed, Nabeel Reza, Yanti Johari, Dasmawati Mohamad, Johari Yap Abdullah, Tahir Yusuf Noorani

**Affiliations:** 1 School of Dental Sciences, Universiti Sains Malaysia, Health Campus, Kubang Kerian, Kota Bharu 16150, Kelantan, Malaysia; 2 Prosthodontic Unit, School of Dental Sciences, Universiti Sains Malaysia, Health Campus, Kubang Kerian, Kota Bharu 16150, Kelantan, Malaysia; 3 Department of Clinical Sciences, Center of Medical and Bio-Allied Health Sciences Research, College of Dentistry, Ajman University, Ajman 346, United Arab Emirates; 4 Department of Operative Dentistry, College of Dentistry, University of Anbar, Baghdad 55431, Iraq; 5 School of Dentistry, Management and Science University, University Drive Off Persiaran Olahraga, Section 13, Shah Alam 40100, Selangor, Malaysia; 6 Biomaterial Unit, School of Dental Sciences, Universiti Sains Malaysia, Health Campus, Kubang Kerian, Kota Bharu 16150, Kelantan, Malaysia; 7 Craniofacial Imaging Laboratory, School of Dental Sciences, Universiti Sains Malaysia, Health Campus, Kubang Kerian, Kota Bharu 16150, Kelantan, Malaysia; 8 Conservative Dentistry Unit, School of Dental Sciences, Universiti Sains Malaysia, Health Campus, Kubang Kerian, Kota Bharu 16150, Kelantan, Malaysia

**Keywords:** novel dental composites, experimental composite resins, dental resin composites, novel dental materials, restorative dentistry

## Abstract

Dental resin composites (DRCs) are widely used restorative materials because of their aesthetics, ease of handling, and compatibility with minimally invasive approaches. Research continues to improve their formulations and properties. This scoping review aimed to summarize and analyze evidence on novel DRCs and their potential development, and to discuss current trends, technologies, and research areas that may shape their evolution. A systematic search of PubMed and Scopus was conducted for studies published or available online from 2010 to 2024. Fifty-nine studies were included in the qualitative synthesis. The included studies described innovations designed to address clinical needs through bioactive materials, antibacterial agents, sustainable fillers, modified monomer systems, and other functional strategies. Future research should optimize additive concentrations, evaluate long-term clinical performance, and address cost-effectiveness, aesthetic limitations, standardization, and translational feasibility.

## 1. Introduction

Dental resin composites (DRCs) are tooth-colored restorative materials used to replace missing enamel and dentin [1]. They are mainly composed of a polymerizable resin-monomer matrix, inorganic filler particles, a silane coupling agent, and a photoinitiator. DRCs are available in different types, mainly packable and flowable [2], and are selected according to material characteristics and clinical function; the different types of flowable composites, in particular, have been described using classifications that reflect their material features and range of clinical uses [2,3]. Clinical applications of DRCs include direct restoration of anterior and posterior teeth, pit and fissure sealants, inlays, onlays, veneers, post-core restorations in endodontically treated teeth, repair of chipped porcelain restorations, and cementation of orthodontic brackets, Maryland bridges, and ceramic crowns [3]. The introduction of resin composites into dentistry dates to the late 1950s and early 1960s [4]. In 1962, bisphenol A-glycidyl methacrylate (Bis-GMA) was developed by Bowen to improve the physical properties of acrylic resins, and composite materials were subsequently produced by incorporating inorganic fillers [4,5]. Since then, DRC formulations and performance have continued to evolve.

DRCs have become widely used alternatives to amalgam restorations because of increasing patient demand for aesthetics, concerns regarding mercury in dental amalgam, ease of manipulation, and compatibility with minimally invasive, tooth-conserving treatment [5]. Contemporary DRCs have improved substantially in formulation and performance, with advances in durability, handling, polymerization shrinkage, physical and mechanical properties, marginal adaptation, and aesthetics. Nevertheless, composite placement remains technique-sensitive, and polymerization shrinkage can still contribute to clinical challenges. Factors associated with restoration failure include poor oral hygiene, inappropriate cavity design, material-related limitations, and manipulation errors. Therefore, research continues to focus on improving DRC performance and longevity [6]. At the same time, interest in sustainable, low-cost, and biocompatible materials has encouraged the exploration of natural biowastes as potential eco-friendly sources for composite fillers.

Novel dental restorative materials are continually being proposed. For this review, novelty was defined as a specified innovation in the composition, structure, source of constituents, or functional mechanism of a DRC. This encompassed experimental formulations, purposeful modifications of commercial composites, and commercial technologies evaluated for an identifiable material innovation. Novelty described the material feature under investigation and did not imply superior performance or clinical effectiveness. Examples include DRCs derived from rice-husk biowaste [7]. More recent work published after the review’s 2010–2024 eligibility window has also evaluated water sorption and solubility in an experimental green-based flowable resin composite, illustrating continued development in sustainability-oriented composite research [8]. Other examples include antimicrobial DRCs containing nano-cerium oxide fillers [9] and low-shrinkage-stress composites containing the antibacterial dimethylaminododecyl methacrylate monomer [10]. A scoping review was therefore considered appropriate to map these diverse developments and identify major research directions.

A scoping review was selected because the evidence base is broad, heterogeneous, and predominantly laboratory-based. The available studies use different formulations, bacterial models, aging protocols, mechanical tests, and outcome measures, making quantitative meta-analysis inappropriate. Unlike a systematic review focused on a narrowly defined intervention and outcome, a scoping review can map a broad range of experimental DRC technologies, describe where the field is concentrated, identify methodological gaps, and clarify which strategies may warrant subsequent systematic reviews or clinical studies.

Accordingly, this review aimed to summarize and analyze the available evidence on novel DRCs and their potential development, and to discuss current trends, technologies, challenges, and research areas that may shape the future evolution of these materials.

## 2. Materials and Methods

This review was conducted in accordance with the Preferred Reporting Items for Systematic Reviews and Meta-Analyses extension for Scoping Reviews (PRISMA-ScR) [11] and was guided by Arksey and O’Malley’s methodological framework for scoping studies [12]. The five-stage approach used in this review is outlined below.

### 2.1. Research Questions

The following research questions guided this scoping review:

1. What advancements and current knowledge exist regarding novel dental composite resins?
2. What challenges have been identified in research on novel dental composite resins?

### 2.2. Search Strategy and Information Sources

Search terms were developed from the relevant literature. A systematic search of PubMed and Scopus was conducted to identify studies for inclusion in this review.

PubMed was selected for its comprehensive biomedical and dental coverage, whereas Scopus was selected as a multidisciplinary database that indexes materials science, engineering, and dental journals. Together, these databases were used to capture both dental- and materials-focused experimental research. The omission of Web of Science, Embase, and other databases is acknowledged as a limitation because relevant studies from adjacent materials science or engineering sources may have been missed. The final searches were conducted on 11 October 2024 in PubMed and 10 July 2024 in Scopus, with eligibility restricted to studies published or available online between 2010 and 2024.

Supplementary records were identified through reference-list screening and expert recommendation. Screening the reference list of the systematic review by Lin et al. [7] identified twelve potentially relevant records, while three additional records were recommended by a research supervisor with expertise in the subject area. The systematic review was used only to identify original research and was not itself included in the qualitative synthesis. All supplementary records were checked for duplicates and assessed against the same eligibility criteria as records retrieved from PubMed and Scopus, with eligibility determined from the original articles.

The search string used for the PubMed database was:

“(novel OR new OR experimental OR original OR innovative OR state-of-the-art OR pioneer) AND ((dental composite) OR (tooth composite) OR (dental resin composite) OR (tooth resin composite) OR (dental flowable composite) OR (tooth flowable composite)) AND ((in vitro study) OR (in vitro experiments) OR (laboratory-based investigations) OR (pilot study) OR (preliminary study)).”

The search string used for the Scopus database was:

“ALL (“novel” OR “new” OR “experimental” OR “original” OR “innovative” OR “state-of-the-art” OR “pioneer”) AND ALL (“dental composite” OR “tooth composite” OR “dental resin composite” OR “tooth resin composite” OR “dental flowable composite” OR “tooth flowable composite”) AND ALL (“in vitro study” OR “in vitro experiments” OR “laboratory-based investigations” OR “pilot study” OR “preliminary study”).”

Eligible reports evaluated novel, new, and/or experimental dental composite resins in in vitro or laboratory-based studies. For articles reporting both laboratory and human intraoral investigations, only the laboratory component was eligible, provided that its methods and results were separately reported and met all other inclusion criteria. Findings from in situ or other human intraoral experiments were excluded from data charting and synthesis. Exclusion criteria comprised randomized and non-randomized controlled clinical trials, prospective and retrospective cohort studies, animal studies, expert opinions, case series, case reports, systematic reviews, and narrative/literature reviews. Methodological or protocol publications that did not directly evaluate an eligible novel DRC formulation were also excluded, as were reports not published in English and rice-husk composite studies unrelated to dental applications.

### 2.3. Study Selection

Studies published or available online between 2010 and 2024 were screened according to the predefined inclusion and exclusion criteria. Study selection followed the PRISMA-ScR reporting framework. After seven duplicates were removed, 1015 records were retained for title and abstract screening. Screening was conducted independently by two reviewers [LKP, MAR], and they independently assigned each study to a primary novelty category. Differences were resolved through discussion and agreement with a third reviewer [TYN].

### 2.4. Data Charting and Qualitative Appraisal

Study characteristics, including authors, year of publication, type of composite resin, type of novelty, formulation and key tests, and main outcomes, were charted from each included article for qualitative synthesis. A formal risk-of-bias assessment was not undertaken; instead, a structured qualitative appraisal was used to support interpretation of the predominantly preclinical evidence. The appraisal considered the use of appropriate controls, sample size or replication, clinically relevant aging or storage protocols, recognized mechanical or biological testing standards, relevance of antibacterial/biofilm models, cytotoxicity or biocompatibility testing, and whether improvements in biological performance were accompanied by compromises in mechanical or aesthetic properties. Appraisal findings were summarized by novelty category in Supplementary Table S1, with supporting study references and their implications for interpreting the evidence.

### 2.5. Synthesis and Reporting

The main categories of DRC novelty were identified after completion of the scoping review, and the findings were collated, summarized, and reported by category in the Results and Discussion sections.

Each study was assigned to one primary category according to its stated principal objective and the material innovation central to that objective. For multifunctional formulations, the intended role of the modified constituent and the experimental comparisons were considered together to determine the primary category. Assignment was not based on the largest or most statistically significant improvement. Studies centered on rice husk-derived constituents were grouped according to their material source, while additional functions were described within the study summaries. Other innovations were reported under their respective themes within the “other novel DRCs” group.

### 2.6. Use of Generative Artificial Intelligence

AI was used during manuscript preparation for language editing. The authors reviewed the scientific content and take responsibility for the final manuscript.

## 3. Results

**Figure 1:**
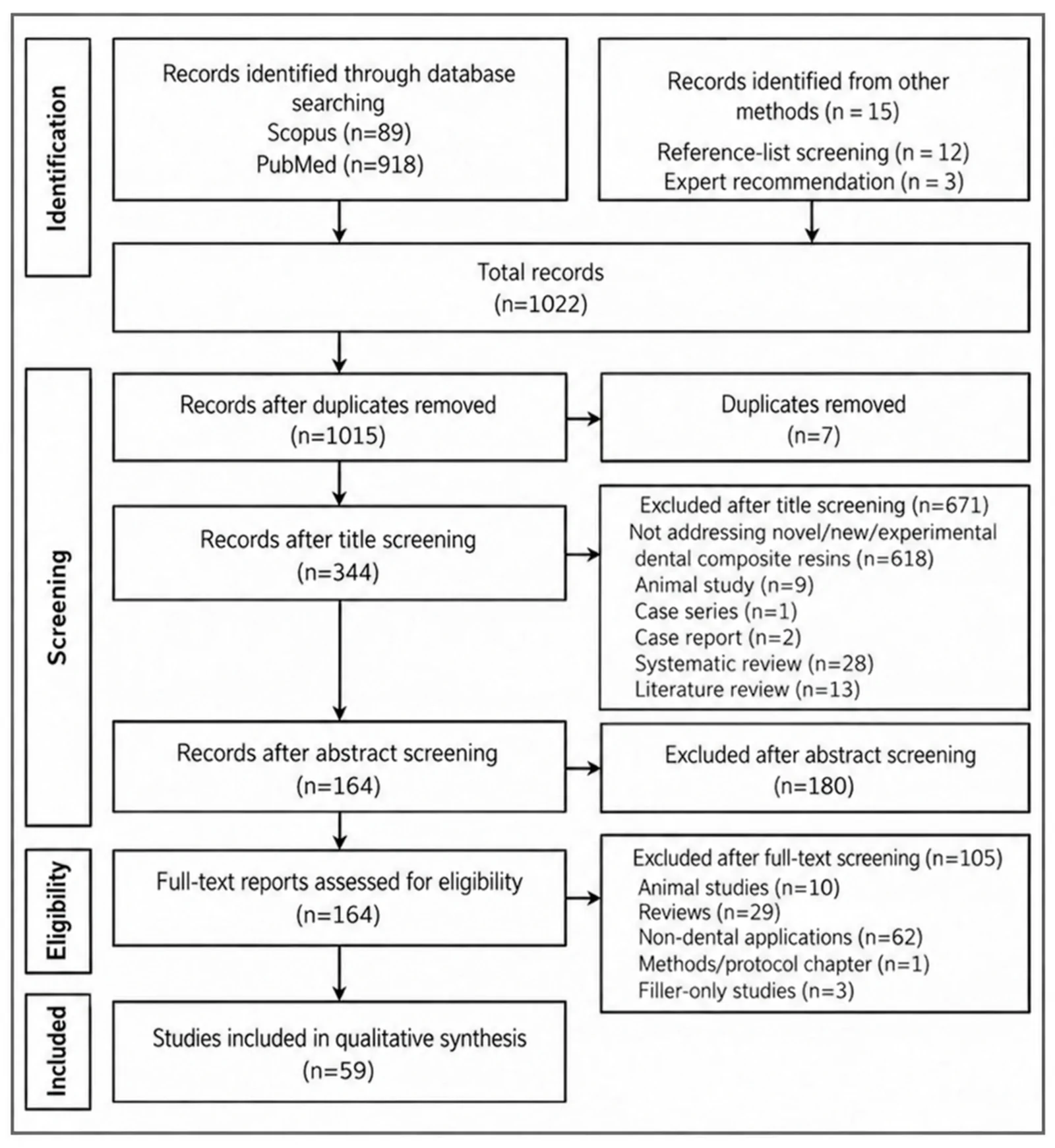
PRISMA-ScR flow diagram of study selection. The diagram summarizes identification, screening, eligibility assessment and inclusion of the 59 studies in the qualitative synthesis.

**Figure 2:**
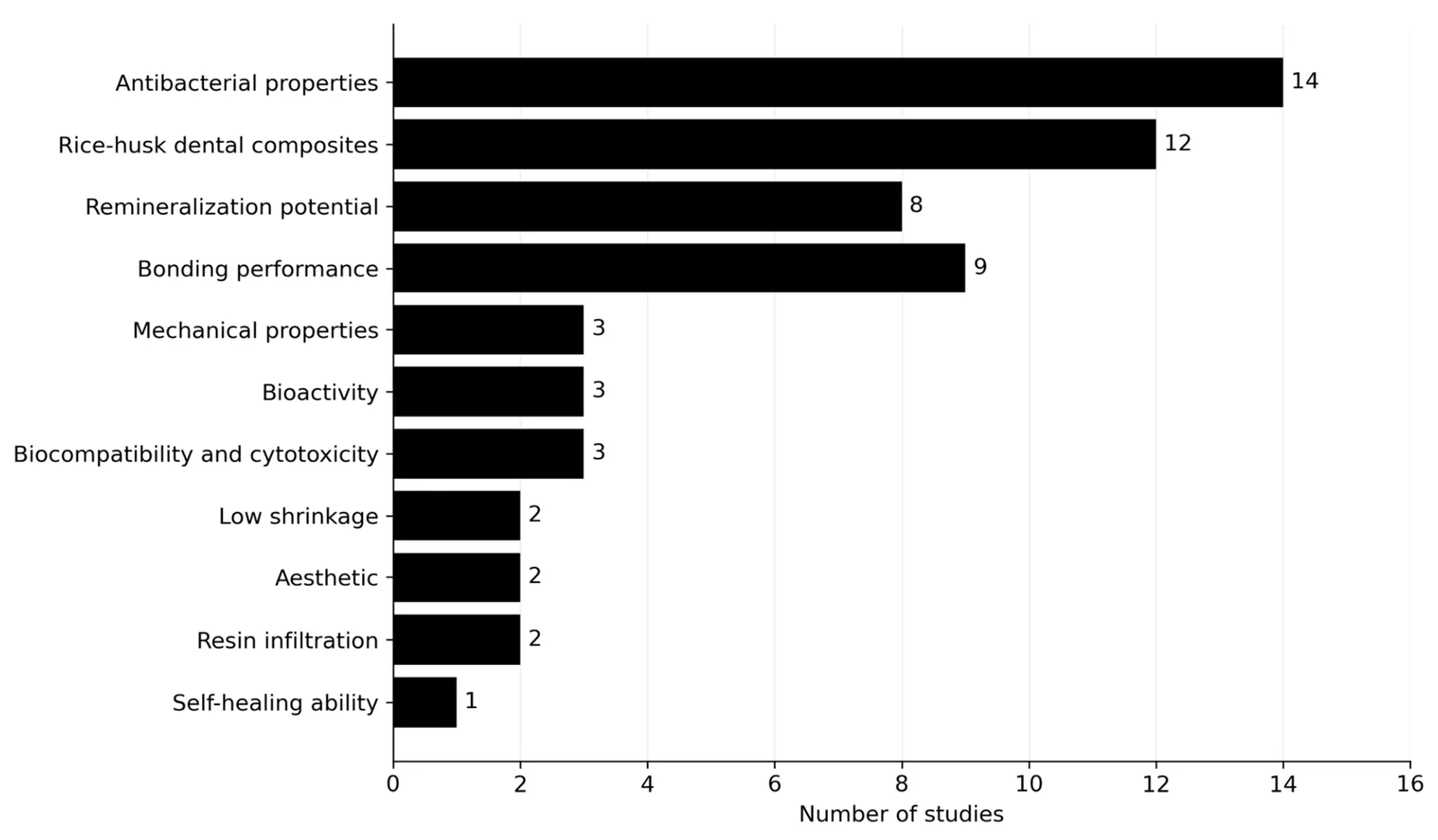
Distribution of the 59 included studies by primary novelty category. Each study was counted once according to its primary assigned category, although individual formulations could have multiple functions. Counts describe the evidence captured by this review and do not indicate the relative importance, effectiveness, or clinical maturity of each research direction.

The search identified 89 records from Scopus and 918 from PubMed, giving 1007 database records. An additional 15 records were identified through supplementary sources: twelve through reference-list screening of the systematic review by Lin et al. [7] and three through recommendation by a research supervisor, resulting in 1022 records before duplicate removal. Seven duplicates were removed, leaving 1015 records for screening. Title screening excluded 671 records, leaving 344 for abstract screening; a further 180 records were excluded at the abstract stage, leaving 164 full-text reports for eligibility assessment. After 105 full-text reports were excluded, including one methods/protocol chapter that did not directly evaluate an eligible novel DRC formulation, 59 studies were included in the qualitative synthesis (Figure 1). The characteristics of the included studies are summarized in Table 1, Table 2, Table 3, Table 4 and Table 5, and Figure 2 summarizes the number of studies by novelty type.

### 3.1. Summary of Included Studies

The 59 included studies were categorized according to their primary type of novelty. Table 1, Table 2, Table 3, Table 4 and Table 5 summarize the study characteristics and key findings. The charted assessments showed recurring category-specific test domains. Antibacterial studies assessed bacterial adhesion, viability, or growth, often alongside material properties. Remineralizing studies assessed mineral recovery or deposition, ion release, and related tooth or material outcomes. Bonding-related studies used bond-strength, leakage, or interfacial assessments, with some also evaluating conversion and mechanical properties. Rice husk-derived composite studies emphasized filler characterization and physical or mechanical properties. The remaining category used application-specific outcomes, including color, gloss, cytotoxicity, infiltration, and self-healing. These domains overlap and represent a descriptive summary of the charted assessments, not an exhaustive frequency ranking of individual tests.

### 3.2. Descriptive Methodological Appraisal

The category-level appraisal is summarized in Supplementary Table S1. Methodological strengths included matched controls, aging protocols, and combined functional and mechanical assessment in selected studies. Limitations included heterogeneous models and test conditions, unclear independent replication in some reports, and limited concurrent durability and biological evaluation. These differences restrict direct comparison across studies and categories.

## 4. Discussion

### 4.1. Patterns Across Material Innovations

The included studies investigated several complementary approaches to DRC development: antibacterial and remineralizing functions, improved bonding and physical performance, alternative filler sources, and other targeted material modifications. Table 6 summarizes and compares the major innovation categories, their key benefits and limitations, and priority evidence gaps. These categories overlap because a single formulation may address several objectives. Category frequencies describe the distribution of the retrieved studies according to their primary assigned innovation; they do not establish the relative importance, effectiveness, or clinical readiness of each approach.

Across the retrieved studies, a common design direction was to add biological or reparative functions to conventional restorative performance. Combining antibacterial monomers with ion-releasing fillers illustrates the development of multifunctional formulations intended to address both biofilm activity and mineral loss [14,20]. Other approaches target different restorative needs: self-adhering systems seek to simplify placement of DRCs [35,37,42,43], self-healing systems aim to repair material damage [71], and resin infiltrants address penetration and filling of carious lesions [66,67]. Rice husk-derived fillers extend innovation to constituent sourcing, although their environmental advantages require separate verification [44,45,46,47,48,49,50,51,52,53,54,55]. These approaches suggest complementary priorities for restorative dentistry, including controlling disease-associated processes, preserving interfaces, maintaining material integrity and improving resource use. They describe design directions within the retrieved evidence, rather than demonstrated temporal trends or established clinical benefits.

**Table 6:** Comparative summary of major innovation strategies.

Strategy	Representative Examples	Key Benefit	Main Limitation	Priority Evidence Gap
Antibacterial monomers/particles	DMAHDM, UDMQA-12, QAUDMAs, QASi, ZnO, Ag-BG, AgBr, PACs	Lower biofilm viability; some effects persisted after short aging	High loading may affect conversion, aesthetics or strength; ion/antibiotic systems require safety monitoring	Long-term multispecies biofilms, saliva/pellicle models and clinical caries outcomes
Remineralizing/bioactive fillers	NACP, DCPD, TTCP, HA, bioactive glass, fluoride-BG	Ca/P release, apatite formation and lesion remineralization	Excessive ion-releasing filler may increase sorption/leaching or reduce strength	Standard recharge/re-release protocols and long-term marginal-sealing studies
Low-shrinkage/self-healing systems	Silorane, polybenzoxazine, silanized PUF microcapsules	Lower shrinkage stress, better marginal behavior or partial crack recovery	Capsules can weaken flexural strength; repair durability needs fatigue validation	Standardized cyclic loading, thermomechanical aging and self-healing methods
Bonding/self-adhesive systems	Self-adhering flowables, nHA-PU, PBO-ZnO-POSS, nanoparticle modification	Simpler handling and selected improvements in bonding/interface performance	Dentin bonding remains variable; hydrophilic/acidic phases may degrade	Standardized substrate handling, recognized bond tests and clinically relevant aging
Sustainable rice-husk DRCs	Rice-husk nanosilica, silanized fillers, zirconia-reinforced hybrids	Potential sustainable filler with acceptable physical/mechanical properties in selected formulations	Processing and quality control may offset raw-material cost and sustainability advantages	Life-cycle analysis, production cost and batch-to-batch reproducibility

### 4.2. Trade-Offs Between Functionality and Material Performance

A recurring issue was whether an additional function could be achieved without compromising the properties required for a durable restoration. Increasing functional additive content may enhance antibacterial activity or mineral formation, but the resulting performance depends on concentration, resin composition, and filler–matrix compatibility. Selected remineralizing formulations showed improvements in mineral-related outcomes alongside reductions in mechanical properties [27,28,29,31]. Conversely, some antibacterial formulations retained the measured mechanical properties under the conditions tested [20,21,24]. These findings support formulation-specific optimization rather than assuming that higher additive concentrations provide greater overall benefit.

A targeted re-examination identified at least seven studies reporting a functional benefit alongside improved mechanical performance or no statistically significant reduction in the measured mechanical or bonding properties for selected formulations [13,14,17,20,21,31,71]. These included antibacterial formulations [13,14,17,20,21], a rechargeable ion-releasing cement [31] and a self-healing composite containing silanized microcapsules [71]. This is a verified minimum of formulation-specific examples, not an exhaustive count of studies without trade-offs. It does not establish absence of aesthetic, biological, or long-term durability compromises, and other formulations within the same studies could perform less favorably. In particular, a non-significant difference does not demonstrate equivalence. An overall proportion of “compromise-free” studies therefore cannot be established from these heterogeneous and incompletely assessed outcomes.

Related trade-offs occur in other categories. Self-healing systems must balance healing capacity against the effect of microcapsules on the original material strength [71]. Resin infiltrants must combine sufficient penetration with the properties needed to fill surface defects [66,67]. For alternative filler sources, satisfactory performance depends on processing, dispersion and interfacial compatibility, as well as the source of the filler itself [44,45,46,47,48,49,50,51,52,53,54,55]. Future comparisons should therefore assess functional benefits and potential mechanical, optical or bonding compromises within the same formulation and after aging. The acceptable balance will also depend on the intended use: a load-bearing restoration, an infiltrant, and a bonding material have different functional requirements. An improvement in one measured property should therefore be interpreted against application-specific demands rather than treated as evidence of overall superiority.

Self-adhering composites aim to simplify placement by reducing separate bonding steps, but fewer steps do not by themselves establish lower technique sensitivity or durable adhesion. The bonding and marginal-integrity studies showed formulation- and substrate-dependent performance [35,36,37,38,39,40,41,42,43]. Durable dentin bonding remains a particular challenge; future evaluations should control tooth-substrate preparation and moisture conditions and assess bond retention and failure modes after aging.

### 4.3. Common Evidence Gaps and Testing Needs

Differences in formulations, controls, specimen preparation, curing conditions and outcome measurements limited direct comparison across the studies, including within the same novelty category. Antibacterial models included single-species *S. mutans* biofilms, a six-species adhesion model and saliva-derived communities, with outcomes such as colony counts, quantitative PCR and growth monitoring [13,14,15,16,22]. Remineralization assessments included apatite deposition, microradiographic mineral recovery and microhardness, which measure different aspects of material or tooth response [27,28,29,33,34]. Bonding-related assessments included shear bond strength, dye leakage and optical imaging of interfaces; protocols ranged from immediate testing to thermocycling or prolonged storage [35,37,40,41,42]. Thus, a shared test-domain label does not imply methodological equivalence. This heterogeneity was described qualitatively; no statistical heterogeneity estimate or pooled effect size was calculated.

The principal methodological need is more comparable testing that also reflects the intended clinical application. Studies should clearly report specimen numbers, independent experimental replicates, curing conditions, and appropriate controls. Functional outcomes should be evaluated alongside mechanical and interfacial performance, using applicable testing standards and clearly justified adaptations. Antibacterial assessments would benefit from consistent reporting of biofilm composition, contact conditions, aging, and the distinction between bacterial adhesion, viability, and growth inhibition. Stronger evidence for a promising formulation would combine a functional benefit over an appropriate comparator with retained mechanical and interfacial performance after aging. Where a study assesses only one of these domains, its findings support further investigation rather than an overall judgment of material suitability. In this review, “promising” denotes a candidate for further investigation under the reported experimental conditions, rather than a validated clinical recommendation.

The standards-reporting audit identified explicit references to named standards in 25 of the 59 studies (42.4%) [13,18,19,20,21,23,24,27,33,37,41,43,44,47,51,53,54,55,57,58,61,63,68,69,71]. No explicit reference was identified for the assessed endpoints in the remaining 34 studies. ISO 4049 was cited in 11 studies [13,21,24,43,44,47,53,54,55,61,68], across different editions and endpoints. ISO 10993-5 was referenced in three studies: two linked it to their cell-extract methods [24,63], whereas one discussed it when interpreting cytotoxicity [69]. Adhesion-related standards included ISO 29022 [27] and ISO/TS 11405 [33,37,41]. These figures quantify reporting, not the proportion fully compliant with a standard. Modified procedures and differences in standard editions prevent an inference of uniform testing, and absence of an explicit citation does not establish that a standard was not followed.

Where applicable, mechanical and physical testing should follow ISO 4049:2019 for polymer-based restorative materials [72], and in vitro cytotoxicity testing should follow ISO 10993-5:2009 [73]. Adhesion testing can use ISO 29022:2013 for notched-edge shear bond strength [74] or ISO/TS 4640:2023 for tensile bond strength to tooth structure [75], according to the research question. These methods should be complemented by application-specific aging protocols; conformity with a laboratory standard alone does not establish clinical effectiveness.

Longer-term assessment is particularly important for functions that depend on sustained activity. Ion release, antibacterial effects, and self-healing should be examined together with property retention after relevant chemical, thermal and mechanical challenges. Similarly, short-term cell viability findings provide limited evidence of biological safety and should be interpreted in relation to the tested cells, concentrations and exposure periods. For emerging functions, comparable protocols should specify ion recharge and re-release cycles, crack generation and healing conditions, and retention of function during repeated challenges. Aesthetic outcomes should also be evaluated after staining, brushing and wear, alongside surface roughness and gloss retention.

### 4.4. Barriers to Clinical Translation

The laboratory evidence identifies candidate materials and mechanisms for further development, but does not establish improvements in clinical restoration longevity, secondary caries prevention or patient satisfaction. Translation requires evidence that functional benefits persist under oral conditions while handling, curing, bonding, wear resistance, and aesthetics remain suitable for the intended application. Potential reductions in repeat treatment, patient burden and costs remain hypotheses that require direct clinical and economic evaluation.

Manufacturing reproducibility and feasibility also require attention. For rice husk-derived fillers, an agricultural waste source does not by itself demonstrate lower environmental impact or production cost. Extraction, purification, surface treatment and quality control must be considered alongside composite performance. Comparative environmental and economic assessments are therefore needed before sustainability advantages can be established.

Similar challenges have been highlighted for zeolite-based dental materials, including the need for long-term biocompatibility testing, clinical validation and compliance with regulatory requirements. Zeolites are being explored as multifunctional fillers with the potential to combine ion release, antibacterial activity and mechanical reinforcement [76].

Progress towards clinical use should combine reproducible formulation and laboratory validation with subsequent clinical evaluation. The priority is to demonstrate a durable overall benefit under relevant conditions, rather than an improvement in an isolated laboratory endpoint.

### 4.5. Study Limitations

The literature search was restricted to PubMed and Scopus. Although these databases provide complementary biomedical and multidisciplinary coverage, relevant studies indexed exclusively in other databases may have been missed. In addition, requiring both novelty-related and laboratory-design descriptors may have excluded eligible experimental studies that did not use these terms in their searchable bibliographic records. Search sensitivity was not formally validated, and the extent of potential omissions remains unknown. These limitations may have affected both the breadth of the evidence map and the distribution of studies across novelty categories. Accordingly, category frequencies describe the studies retrieved and included in this review and should not be interpreted as measures of the relative importance or prevalence of research directions across the entire field.

Additional limitations of the evidence base include small or unreported sample sizes, inconsistent use of commercial and experimental controls, limited thermocycling or water-storage protocols, short-term antibacterial testing, single-species bacterial models that do not fully simulate oral biofilms, variable cytotoxicity assays, and inconsistent reporting of filler dispersion and degree of conversion. These differences limit direct comparison of effect sizes across novelty categories. The findings should therefore be interpreted as a map of promising research directions rather than definitive evidence of clinical superiority.

The descriptive appraisal cannot establish the risk of bias in individual studies or provide a definitive ranking of material strategies. Small or unreported sample sizes, limited aging and restricted biological models reduce confidence that observed benefits are reproducible or transferable to clinical use. Accordingly, the findings should guide further testing rather than selection of materials for clinical practice.

## 5. Conclusions

The studies retrieved for this scoping review, published or available online from 2010 to 2024, investigated antibacterial and remineralizing functions, rice husk-derived fillers, bonding-related strategies and other material innovations. These approaches may address important causes of restoration failure, including secondary caries, marginal degradation, polymerization shrinkage, poor dentin bonding, and material wear. However, most evidence remains laboratory-based. Before novel formulations can be recommended for routine clinical use, future research should demonstrate durable mechanical properties, reliable dentin bonding, stable aesthetics, acceptable radiopacity and polishability, biocompatibility, clinically meaningful antibacterial or remineralizing effects, and economic and environmental feasibility. Standardized testing followed by well-designed long-term clinical studies will be essential for translating promising experimental formulations into restorative materials with demonstrated patient benefit and restoration longevity.

## Figures and Tables

**Table 1:** Summary of studies investigating material strategies intended to confer antibacterial functionality.

Author	Year	Composite/Strategy	Formulation and Basis of Novelty	Main Assessments	Key Finding
Zhou et al. [13]	2024	Rod-like ZnO-modified DRC	Experimental composition: Bis-GMA/TEGDMA + 2.5–7.5 wt% rod-like ZnO	Morphology; mechanical properties; *S. mutans*; HGF-1 cytotoxicity	All ZnO levels were antibacterial and non-cytotoxic; 5 wt% gave the best strength and ~40% biofilm inhibition.
Zhou et al. [14]	2020	DMAHDM/NACP bioactive DRC	Experimental multifunctional formulation: Bis-GMA/TEGDMA + 3% DMAHDM and/or 30% NACP	Flexural properties; saliva biofilm; Ca/P release; enamel remineralization	DMAHDM reduced biofilm (~2 log); NACP increased acid-triggered Ca/P release; the combination restored enamel hardness close to that of sound enamel.
Zhang et al. [15]	2021	PAC-modified adhesive	Modified adhesive resin: covalently incorporated penicillin V- or ciprofloxacin-based polymer-antibiotic conjugates	*S. mutans* biofilm; 30-day salivary-enzyme aging	Sustained antibacterial activity and reduced adhesion; PV-PAC retained antibiofilm activity after aging.
Henrich et al. [16]	2016	Antibacterial monomer DRC	Experimental monomer modification: 4% chlorinated antibacterial methacrylate incorporated into resin composite	In vitro mixed-species bacterial adhesion on saliva-conditioned composite discs; TaqMan qPCR after 8 and 24 h	Early attachment fell to 58.6% of control at 8 h and 92.6% at 24 h, with reduced *A. viscosus* and *S. mitis* colonization.
Chatzistavrou et al. [17]	2015	Ag-BG bioactive DRC	Modified commercial composite: Tetric EvoFlow + 1, 5, or 15 wt% silver-doped bioactive glass for combined functional effects	Antibacterial activity; apatite formation; pH; bond strength	5 and 15 wt% Ag-BG inhibited *S. mutans* and *E. coli*; hydroxyapatite formed by day 3 without bond-strength loss.
Zamanian et al. [18]	2010	Ca(OH)2/nHA mouldable DRC	Experimental cement composition: Ca(OH)_2_/nHA powder (19–56 wt% Ca(OH)_2_) + salicylate liquid	Setting; compressive strength; ion release; pH; antibacterial activity	More Ca(OH)_2_ increased pH, Ca release and antibacterial activity; compressive strength was 8–16 MPa.
Chroszcz et al. [19]	2021	QAUDMA-containing DRC	Monomer precursor innovation: QAUDMAs with C8-C18 alkyl chains	Conversion; shrinkage; viscosity; thermal and optical properties	Conversion was 53–79% and shrinkage 1.24–2.99%; optical properties matched natural enamel.
Albeshir et al. [20]	2022	Dual-function flowable DRC	Experimental matrix/additive combination: UDMA/TEG-DVBE + 5% DMAHDM + 20% NACP + 30% glass for antibacterial and ion-releasing functions	Mechanical/flow properties; roughness; biofilm inhibition	Mechanical properties remained acceptable; DMAHDM reduced biofilm bacteria by 3–6 logs and lowered metabolic activity.
Cao et al. [21]	2017	AgBr/BHPVP nanocomposite	Functional filler innovation: <1 wt% core-shell AgBr/cationic polymer in dental resin	Mechanical properties; Ag^+^ release; *S. mutans*; cytotoxicity	Improved hardness without mechanical loss and provided long-term antibacterial activity with good biocompatibility below 1 wt%.
Dekel-Steinkeller et al. [22]	2022	QASi bulk-fill flowable DRC	Functional filler innovation: Infinix flowable composite + quaternary ammonium silica filler	In vitro direct-contact test against *E. faecalis* and pooled whole-saliva bacteria after seven days of PBS aging; optical-density growth monitoring	No detectable growth of *E. faecalis* or saliva-derived bacteria following direct contact with the QASi-containing composite under the test conditions.
Weng et al. [23]	2012	Furanone-containing DRC	Experimental monomer modification: DRC + 5–30% furanone derivative	Compressive strength; *S. mutans* viability	Maintained compressive strength and reduced *S. mutans* viability by 16–68%.
Huang et al. [24]	2018	UDMQA-12-modified DRC	Experimental antibacterial monomer: Bis-GMA/TEGDMA + 9 wt% UDMQA-12 + 70 wt% glass	Antibacterial activity; cytotoxicity; flexural properties	Antibacterial activity persisted after water/saliva exposure; mechanical properties met ISO requirements with low cytotoxicity.
Zhang et al. [25]	2019	Ciprofloxacin-PAC DRC	Modified adhesive resin: Cip-HEMA PAC in adhesive resin (1.84 wt% effective antibiotic) for antibiotic delivery	Antibiotic release; *S. mutans*; morphology; conversion	Maintained conversion and released antibiotic over 25 days (5.12%); bacterial morphology was damaged.
Yang et al. [26]	2014	MAE-DB/Portland cement DRC	Experimental functional combination: Light-cured composite + Portland cement + 5 wt% MAE-DB	*S. mutans* antibacterial activity	Strong antibacterial activity against *S. mutans*.

Footnote: Classification in this category reflects the intended antibacterial role of the material innovation and does not necessarily indicate that antibacterial activity was experimentally demonstrated. The assessments and findings reported in each row should be interpreted according to the tests performed; material characterization alone does not establish antibacterial efficacy.

**Table 2:** Summary of studies testing the remineralizing novel DRCs.

Author	Year	Composite/Strategy	Formulation and Basis of Novelty	Main Assessments	Key Finding
Lone et al. [27]	2024	Polylysine/calcium-phosphate DRC	Experimental functional combination: UDMA/PPGDMA + silica + MCPM + β-TCP + 0–10 wt% polylysine	Conversion; mechanical properties; apatite formation	Conversion remained 68–72%; additives reduced mechanical properties, while 10 wt% polylysine produced the densest apatite layer.
Alania et al. [28]	2019	DCPD-containing DRC	Experimental mineral-releasing filler: Bis-GMA/TEGDMA + 0, 10, or 20 vol% DCPD within 60 vol% filler	pH cycling; transverse microradiography	DCPD improved enamel mineral recovery and reduced lesion depth.
Weir et al. [29]	2017	NACP/TTCP nanocomposite	Experimental mineral-releasing formulation: EBPADMA/PMGDM + 40% NACP, with or without 20% TTCP	Flexural properties; Ca/P release; dentin remineralization	Acidic conditions increased Ca/P release; dentin remineralization reached 48.2% after 8 weeks.
Mohn et al. [30]	2024	Bioactive-glass DRC	Experimental bioactive fillers: BG 45S5 or fluoride-containing BG incorporated into DRC	Marginal integrity after 10,000 thermocycles and saliva aging	BG-containing DRC used with adhesive showed better aged marginal integrity than the commercial reference.
Xie et al. [31]	2017	Rechargeable NACP orthodontic cement	Experimental recharge function: PMGDM/EBPADMA + 40% NACP for Ca/P recharge and re-release	Bond strength; Ca/P release; recharge/re-release	Bond strength matched commercial cement; one recharge sustained Ca/P release for 14 days.
Tezvergil-Mutluay et al. [32]	2017	Bioactive-glass DRC	Experimental bioactive filler: 50 wt% BG 45S5 or fluoride-containing BAG-F for dentin-interface mineralization	Dentin remineralization; collagen degradation; mechanical properties	Both promoted remineralization and pH > 9; BAG-F reduced collagen degradation and increased dentin modulus by 13%.
Rahmanpanah et al. [33]	2023	nHA-containing DRC	Experimental mineral-releasing formulation: Bis-GMA/TEGDMA + 2, 5, or 10 wt% nHA	Bond strength; ARI; microhardness; remineralization	5–10 wt% nHA improved remineralization; 10 wt% reduced bond strength, although all groups remained acceptable.
Weir et al. [34]	2012	NACP nanocomposite	Experimental remineralizing filler: NACP-containing DRC for Ca/P delivery	30-day pH cycling; enamel microradiography	Enhanced enamel mineral recovery versus fluoride-releasing and untreated controls.

**Table 3:** Summary of studies testing the bonding performance of novel DRCs.

Author	Year	Composite/Strategy	Formulation and Basis of Novelty	Main Assessments	Key Finding
Park et al. [35]	2015	Self-adhering flowable DRC	Experimental self-adhering technology: EF vs. PLP + Filtek Supreme XT Flowable	SS-OCT interfacial defects; 100-day water storage	EF had more enamel-margin defects; PLP had more dentin-margin defects; water storage increased enamel defects in both.
Alb et al. [36]	2010	Experimental DRC (C1)	Experimental C1 resin/Ad 1 adhesive system: Class II/V restorations with C1 + total-etch adhesive	SEM interface; dye penetration	Interface quality was lower, leakage was similar to that of commercial systems, and gingival margins leaked more than enamel margins.
Brueckner et al. [37]	2017	Self-adhering flowable DRCs	Experimental and commercial self-adhering technologies: Vertise Flow, Fusio Liquid Dentin, experimental self-adhering composite vs. conventional adhesive	Microleakage; SEM; shear bond strength; thermocycling	Conventional adhesive systems had higher bond strength; self-adhering materials showed variable marginal sealing.
Hu et al. [38]	2022	PBO fiber-reinforced DRC	Experimental reinforcement structure: PBO fibers modified with ZnO nanoparticles/nanowires and POSS to improve resin coupling	Interfacial shear strength; flexural properties; cytotoxicity	PBO-ZnO NWs-POSS gave the highest interfacial shear strength (29.31 MPa) and flexural strength (925 MPa), with acceptable cytotoxicity.
Khan et al. [39]	2012	nHA-polyurethane bioactive DRC	Experimental root-filling composition: Polyurethane composite + covalently bonded nHA for bioactive interfacial bonding	Chemical structure; apatite formation; dentin push-out bond	nHA promoted a bioactive layer and significantly improved dentin bond strength versus the commercial control.
Nikpour et al. [40]	2021	Nanoparticle-modified self-adhesive DRC	Modified commercial composite: Vertise Flow + TiO_2_, SiO_2_, or TiO_2_/SiO_2_ (0.5–1%)	Shear bond strength; ARI	Nanoparticles slightly improved bonding; 1% TiO_2_ produced the highest shear bond strength with acceptable bonding.
Sterzenbach et al. [41]	2015	Self-adhesive resin cement core	Experimental self-adhesive resin cement: Dual-curing SARC under different curing protocols vs. chemical-cure control	12-month aging; crack propagation/crown survival	Auto- and dual-cured SARC increased crack propagation and crown failure; open-matrix SARC improved survival.
Goracci et al. [42]	2013	Self-adhesive flowable DRC	Commercial self-adhesive technology: Vertise Flow with/without phosphoric acid vs. conventional orthodontic systems	Shear bond strength; ARI; thermocycling	Initial bonding was acceptable but declined after aging; adhesive failure increased after thermocycling.
Czasch et al. [43]	2013	Self-adhesive flowable DRC	Commercial functional technologies: Vertise Flow vs. low-shrinkage and conventional flowable composites	Conversion; hardness; flexural properties; curing depth	Performance depended on filler and curing; Vertise Flow was reliable with 40 s curing and ≤2 mm increments.

**Table 4:** Summary of studies testing the rice husk-derived novel DRCs.

Author	Year	Composite/Strategy	Formulation and Basis of Novelty	Main Assessments	Key Finding
Noushad et al. [44]	2013	Rice-husk nanosilica DRC	Experimental filler-source substitution: Rice-husk-derived nanosilica incorporated into DRC	Flexural strength and modulus	Flexural strength was 82 MPa and modulus 6.8 GPa.
Yusoff et al. [45]	2018	Flowable rice-husk nanohybrid DRC	Experimental filler-source formulation: 50 wt% rice-husk silica with different Bis-GMA/TEGDMA ratios	Hardness; morphology	Hardness was comparable to commercial flowables; more TEGDMA improved hardness.
Yusoff et al. [46]	2019	Flowable rice-husk nanohybrid DRC	Experimental filler-source formulation: EF50B, EF60B and EF70B in Bis-GMA/TEGDMA resin	Flexural properties; conversion; sorption/solubility; filler distribution	Mechanical properties were comparable to commercial controls with uniform filler dispersion.
Noushad et al. [47]	2016	Rice-husk nanohybrid DRC	Filler-process innovation: Rice-husk silica at filler/matrix ratios of 40/60 or 50/50	Flexural/compressive properties; hardness; roughness	The 50/50 formulation performed best: 107 MPa flexural strength, 191 MPa compressive strength and 39 HV1 hardness.
Ismail et al. [48]	2020	Zirconia-reinforced rice-husk DRC	Experimental hybrid filler: Rice-husk nanosilica + 3 or 5 wt% zirconia	Hardness; compressive strength; conversion; microstructure	Zirconia improved hardness and compressive strength; conversion fell slightly but remained >60%.
Raja et al. [49]	2019	Rice-husk nanocomposite	Experimental filler-source substitution: Rice-husk-derived nanosilica composite	Color stability after coffee/tea exposure	Color stability remained acceptable after beverage exposure.
Lin et al. [50]	2020	Zirconia-reinforced rice-husk DRC	Experimental hybrid filler: Rice-husk nanosilica + 3 or 5 wt% zirconia	Fracture strength; SEM filler distribution	Zirconia increased fracture resistance; 5 wt% was comparable to Filtek Z250.
Lin et al. [51]	2020	Zirconia-reinforced rice-husk DRC	Experimental hybrid filler: Rice-husk nanosilica + 3 or 5 wt% zirconia	Polymerization shrinkage; degree of conversion	Zirconia reduced shrinkage while maintaining clinically acceptable conversion.
Bhat et al. [52]	2017	Rice-husk nanosilica DRC	Experimental filler-source substitution: Bis-GMA/TEGDMA resin + 0–3 wt% silane-treated 20 nm nanosilica	Simulated dental wear	3 wt% nanosilica gave the highest wear resistance and reduced surface damage.
Al-Rawas et al. [53]	2021	Flowable rice-husk DRC	Experimental filler-source formulation: EFC50B and EFC40B vs. commercial control	Sorption/solubility; conversion; hardness; morphology	Water sorption was higher than in the control; optimal curing was 40 s for EFC40B and 80 s for EFC50B.
Azlisham et al. [54]	2025 (online first: 2024)	Zirconia/rice-husk nanohybrid DRC	Experimental radiopaque filler/matrix combination: Rice-husk silica + zirconia with UDMA/Bis-GMA ratios from 20:80 to 90:10	Flow; radiopacity; cure; roughness; shrinkage; compression	More UDMA improved strength and cure depth and reduced shrinkage/roughness; performance was comparable to commercial controls.
Azlisham et al. [55]	2023	UDMA-based rice-husk flowable DRC	Experimental matrix substitution: Rice-husk nanohybrid silica with UDMA/Bis-GMA ratios of 40:60–60:40	Conversion; cure depth; hardness; roughness	UDMA-based formulations showed higher conversion (60–65%), greater cure depth and hardness; roughness was comparable.

**Table 5:** Summary of studies reporting other novel DRCs.

Author	Year	Focus	Composite/Strategy	Formulation and Basis of Novelty	Main Assessments	Key Finding
Odermatt et al. [56]	2020	Bioactivity	Bioactive-glass DRC	Modified composite filler structure: Flowable composite + 15 wt% micro-, nano- or hybrid BG	pH; hydroxyapatite; conversion; hardness	Nano- and hybrid BG rapidly raised pH and promoted dense hydroxyapatite; nano-BG maintained conversion and hardness.
Travan et al. [57]	2010	Bioactivity	Polysaccharide-modified thermoset	Experimental surface functionalization: Bis-GMA/TEGDMA + chitlac or RGD-chitlac coating	Coating; mechanics; durability; osteoblast-like cells	Strength fell ~17–18%; RGD-chitlac increased cell proliferation and bioactivity.
Abdulmajeed et al. [58]	2014	Bioactivity	Bioactive-glass composite	Experimental bioactive interface: 50% Bis-GMA/TEGDMA polymer + 50% BG vs. polymer and BG controls	Clotting; platelet adhesion; fibroblast proliferation	Bioactive-glass composite improved blood compatibility, platelet activation and fibroblast proliferation versus the polymer control.
Pastrav et al. [59]	2024	Mechanical	Nanohybrid DRC	Experimental hybrid filler: Experimental PS2 vs. Clearfil Majesty ES-2 and Harmonize	Filler morphology; roughness; compression; color	Clearfil Majesty ES-2 and PS2 showed higher compressive strength and wear resistance.
Thorat et al. [60]	2014	Mechanical	Coupling-agent-free DRC	Experimental reinforcement architecture: Bis-GMA/TEGDMA + 50 wt% nanoporous alumina; Ag-loaded fillers for release	Mechanical stability; aging; Ag release	Mechanical interlocking achieved ~5 GPa storage modulus, improved aging stability and sustained Ag release.
Wang et al. [61]	2015	Mechanical	Bimodal silica DRC	Experimental filler architecture: Silica nanoparticles + silica nanoclusters	Flexural properties; shrinkage; morphology; cytotoxicity	Improved flexural strength/modulus and reduced shrinkage with good biocompatibility.
Sivakumar et al. [62]	2016	Low shrinkage	Silorane-based DRC	Commercial alternative polymerization: silorane-based Filtek P90 LS vs. methacrylate nanohybrid/microhybrid composites	Microleakage after 3000 thermocycles	Occlusal leakage was similar; silorane composite showed lower gingival leakage than methacrylate groups.
Lotfi et al. [63]	2018	Low shrinkage	Polybenzoxazine/zirconia DRC	Experimental matrix formulation: Polybenzoxazine + 60 wt% micro-ZrO_2_ + 10 wt% nano-ZrO_2_ (optimal)	Shrinkage; hardness; modulus; morphology; cytotoxicity	Optimized zirconia increased hardness/modulus and reduced shrinkage; excess zirconia caused agglomeration and poorer mechanics.
Jasse et al. [64]	2013	Aesthetics	Aluminum-barium silicate DRC	Experimental filler-loading series using 40–75% polygonal aluminum-barium silicate filler	Gloss before/after simulated brushing	Higher filler loading improved baseline gloss and gloss retention after abrasion.
Braun et al. [65]	2013	Aesthetics	Aesthetic DRC	Commercial optimal-system innovation: Amaris vs. Arabesk and Synergy	Color stability after water storage/thermocycling	Amaris showed the least color change and better masking of dark backgrounds.
Askar et al. [66]	2018	Resin infiltration	Micro-filled infiltrant	Modified infiltration formulation: MFIR (55% infiltrant + 45% prepolymerized filler) vs. infiltrant and infiltrant + flowable composite	Enamel penetration; cavity filling by CLSM	MFIR maintained enamel penetration and improved cavity filling to ~100% in micro-/cavitated lesions.
Paris et al. [67]	2010	Resin infiltration	Experimental infiltrants	Experimental infiltrant formulations: PC63, PC185, PC204, PC391 vs. flowable and untreated controls	Lesion progression over 400 days	Infiltrants with penetration coefficient > 200 cm/s (PC204, PC391) inhibited lesion progression.
Roman et al. [68]	2016	Biocompatibility	Experimental bioactive DRCs	Experimental hybrid formulations: PM, P14M and P2S with Bis-GMA/UDMA/TEGDMA and hybrid fillers	Sorption/solubility; oral MSC cytotoxicity	Experimental composites showed good physical stability and no cytotoxicity or cytoskeletal disruption.
Chadda et al. [69]	2016	Biocompatibility	HA-filled acrylate DRCs	Experimental bioactive filler composition: Bis-GMA/TEGDMA + 30/50 wt% HA or silica/HA fillers	Cytotoxicity; morphology; cell attachment	All groups supported MSC proliferation; silica/HA improved cell spreading.
Oliveira et al. [70]	2014	Biocompatibility	DMPOH-containing DRC	Experimental initiator-system substitution: Bis-GMA/TEGDMA + 0.2 wt% DMPOH or EDAB	Degree of conversion; cytotoxicity	DMPOH and EDAB gave similar conversion; both reduced cell viability after 24 h exposure.
Rozza et al. [71]	2024	Self-healing	Silanized microcapsule DRC	Experimental self-healing architecture: 0–10 wt% MPS-silanized PUF microcapsules containing TEGDMA + DHEPT	Morphology/chemistry; flexural properties; fracture/self-healing	Silanization preserved flexural strength and improved healing to 49–77% vs. 38–69% without silane; modulus and toughness were unchanged.

## Data Availability

No new data were created or analyzed in this study. Data sharing is not applicable to this article.

## Supplementary Materials

The following supporting information can be downloaded at: https://www.mdpi.com/article/10.3390/polym18202516/s1, Table S1: Category level methodological appraisal.

## Abbreviations

The following abbreviations are used in this manuscript:

Ag	Silver
Ag^+^	Silver ion
Ag-BG	Silver-doped bioactive glass
AgBr	Silver bromide
ARI	Adhesive Remnant Index
BAG-F	Fluoride-containing bioactive glass
BG	Bioactive glass
BHPVP	Brominated and 2-methacryloyloxyethyl functionalized polyvinylpyrrolidone
Bis-GMA	Bisphenol A-glycidyl methacrylate
Ca/P	Calcium/phosphate
Cip	Ciprofloxacin
Cip-HEMA	Ciprofloxacin–2-hydroxyethyl methacrylate conjugate
CLSM	Confocal laser scanning microscopy
DCPD	Dicalcium phosphate dihydrate
DHEPT	N,N-Dihydroxyethyl-p-toluidine
DMAHDM	Dimethylaminohexadecyl methacrylate
DMPOH	4-(N,N-Dimethylamino)phenethyl alcohol
DRC/DRCs	Dental resin composite/dental resin composites
EBPADMA	Ethoxylated bisphenol A dimethacrylate
EDAB	Ethyl 4-(dimethylamino)benzoate
HA	Hydroxyapatite
HEMA	2-Hydroxyethyl methacrylate
HGF-1	Human gingival fibroblast-1
ISO	International Organization for Standardization
ISO/TS	International Organization for Standardization/Technical Specification
MAE-DB	2-Methacryloxylethyl dodecyl methyl ammonium bromide
MCPM	Monocalcium phosphate monohydrate
MFIR	Micro-filled infiltrant resin
MPS	γ-Methacryloxypropyl trimethoxysilane
MSC/MSCs	Mesenchymal stem cell/mesenchymal stem cells
NACP	Nanoparticles of amorphous calcium phosphate
nHA	Nano-hydroxyapatite
PAC/PACs	Polymer–antibiotic conjugate/polymer–antibiotic conjugates
PBO	Poly(p-phenylene-2,6-benzobisoxazole)
PMGDM	Pyromellitic glycerol dimethacrylate
POSS	Polyhedral oligomeric silsesquioxane
PPGDMA	Polypropylene glycol dimethacrylate
PRISMA-ScR	Preferred Reporting Items for Systematic Reviews and Meta-Analyses extension for Scoping Reviews
PUF	Poly(urea-formaldehyde)
PV-PAC	Penicillin V-based polymer–antibiotic conjugate
QAUDMA/QAUDMAs	Quaternary ammonium urethane dimethacrylate/quaternary ammonium urethane dimethacrylates
QASi	Quaternary ammonium silica
qPCR	Quantitative polymerase chain reaction
RGD	Arginine–glycine–aspartic acid peptide sequence
rRNA	Ribosomal ribonucleic acid
SARC	Self-adhesive resin cement
SEM	Scanning electron microscopy
SiO_2_	Silicon dioxide
SS-OCT	Swept-source optical coherence tomography
TEG-DVBE	Triethylene glycol divinylbenzyl ether
TEGDMA	Triethylene glycol dimethacrylate
TiO_2_	Titanium dioxide
TTCP	Tetracalcium phosphate
UDMA	Urethane dimethacrylate
UDMQA-12	Urethane dimethacrylate quaternary ammonium compound with a 12-carbon alkyl chain
ZnO	Zinc oxide
ZrO_2_	Zirconium dioxide
β-TCP	Beta-tricalcium phosphate

## References

[1] Bhattacharya M., Seong W.J., Subramani K., Ahmed W., Hartsfield J.K. (2013). Carbon nanotube-based materials—Preparation, biocompatibility, and applications in dentistry. Nanobiomaterials in Clinical Dentistry.

[2] Al-Rawas M., Yusoff N.M., Johari Y., Mohamad D., Husein A. (2022). The versatility of flowable composites. Part 1: Theory and new classification. Dent. Update.

[3] Al-Rawas M., Yusoff N.M., Johari Y., Mohamad D., Husein A. (2022). The versatility of flowable composites. Part 2: Clinical uses. Dent. Update.

[4] Zhou X., Huang X., Li M., Peng X., Wang S., Zhou X., Cheng L. (2019). Development and status of resin composite as dental restorative materials. J. Appl. Polym. Sci..

[5] Cangul S., Adiguzel O. (2017). The latest developments related to composite resins. Int. Dent. Res..

[6] Milnar F.J. (2011). The evolution of direct composites. Compend. Contin. Educ. Dent..

[7] Lin G.S.S., Cher C.Y., Cheah K.K., Noorani T.Y., Ismail N.H., Ghani N.R.N.A. (2022). Novel dental composite resin derived from rice husk natural biowaste: A systematic review and recommendation for future advancement. J. Esthet. Restor. Dent..

[8] Gan M.J., AL-Jobory A., Fatalla A.A., AL-Rawas M., Johari Y., Othman N.H., Abdullah J.Y., Noorani T.Y. (2025). The effects of different camphorquinone concentrations on the water sorption and solubility of an experimental green-based flowable resin composite. J. Nanostruct..

[9] Varghese E.J., Sihivahanan D., Venkatesh K.V. (2022). Development of novel antimicrobial dental composite resin with nano cerium oxide fillers. Int. J. Biomater..

[10] Alhussein A., Alsahafi R., Wang X., Mitwalli H., Filemban H., Hack G.D., Oates T.W., Sun J., Weir M.D., Xu H.H.K. (2023). Novel dental low-shrinkage-stress composite with antibacterial dimethylaminododecyl methacrylate monomer. J. Funct. Biomater..

[11] Tricco A.C., Lillie E., Zarin W., O’Brien K.K., Colquhoun H., Levac D., Moher D., Peters M.D.J., Horsley T., Weeks L. (2018). PRISMA extension for scoping reviews (PRISMA-ScR): Checklist and explanation. Ann. Intern. Med..

[12] Arksey H., O’Malley L. (2005). Scoping studies: Towards a methodological framework. Int. J. Soc. Res. Methodol..

[13] Zhou S., Liu R., Ma X., Xie Y., Xu X., Du Q., Zhou Z. (2024). Antibacterial effect of novel dental resin composites containing rod-like zinc oxide. Nanotechnol. Rev..

[14] Zhou W., Peng X., Zhou X., Bonavente A., Weir M.D., Melo M.A.S., Imazato S., Oates T.W., Cheng L., Xu H.H.K. (2020). Novel nanocomposite inhibiting caries at the enamel restoration margins in an in vitro saliva-derived biofilm secondary caries model. Int. J. Mol. Sci..

[15] Zhang Z., Jones M.M., Sabatini C., Vanyo S.T., Yang M., Kumar A., Jiang Y., Swihart M.T., Visser M.B., Cheng C. (2021). Synthesis and antibacterial activity of polymer-antibiotic conjugates incorporated into a resin-based dental adhesive. Biomater. Sci..

[16] Henrich B., Hermann I., Di Giulio M., Köhrer K., Deenen R., Sivalingam S., Peters U., Beikler T., Janda R., Rüttermann S. (2016). Reexamination in vitro and in situ of an antibacterially modified experimental dental resin composite with molecular methods: A pilot study. Adv. Mater. Sci. Eng..

[17] Chatzistavrou X., Velamakanni S., DiRenzo K., Lefkelidou A., Fenno J.C., Kasuga T., Boccaccini A.R., Papagerakis P. (2015). Designing dental composites with bioactive and bactericidal properties. Mater. Sci. Eng. C.

[18] Zamanian A., Moztarzadeh F., Kordestani S., Hesaraki S., Tahriri M.R. (2010). Novel calcium hydroxide/nanohydroxyapatite composites for dental applications: In vitro study. Adv. Appl. Ceram..

[19] Chrószcz M.W., Barszczewska-Rybarek I.M. (2021). Synthesis and characterization of novel quaternary ammonium urethane-dimethacrylate monomers—A pilot study. Int. J. Mol. Sci..

[20] Albeshir E.G., Balhaddad A.A., Mitwalli H., Wang X., Sun J., Melo M.A.S., Weir M.D., Xu H.H. (2022). Minimally-invasive dentistry via dual-function novel bioactive low-shrinkage-stress flowable nanocomposites. Dent. Mater..

[21] Cao W., Zhang Y., Wang X., Chen Y., Li Q., Xing X., Xiao Y., Peng X., Ye Z. (2017). Development of a novel resin-based dental material with dual biocidal modes and sustained release of Ag+ ions based on photocurable core-shell AgBr/cationic polymer nanocomposites. J. Mater. Sci. Mater. Med..

[22] Dekel-Steinkeller M., Weiss E.I., Samovici T.L.D., Abramovitz I. (2022). Antibacterial performance of composite containing quaternary ammonium silica (QASi) filler—A preliminary study. J. Dent..

[23] Weng Y., Howard L., Guo X., Chong V.J., Gregory R.L., Xie D. (2012). A novel antibacterial resin composite for improved dental restoratives. J. Mater. Sci. Mater. Med..

[24] Huang Q., Huang S., Liang X., Qin W., Liu F., Lin Z., He J. (2018). The antibacterial, cytotoxic, and flexural properties of a composite resin containing a quaternary ammonium monomer. J. Prosthet. Dent..

[25] Zhang R., Jones M.M., Moussa H., Keskar M., Huo N., Zhang Z., Visser M.B., Sabatini C., Swihart M.T., Cheng C. (2019). Polymer-antibiotic conjugates as antibacterial additives in dental resins. Biomater. Sci..

[26] Yang Y., Huang L., Dong Y., Zhang H., Zhou W., Ban J., Wei J., Liu Y., Gao J., Chen J. (2014). In vitro antibacterial activity of a novel resin-based pulp capping material containing the quaternary ammonium salt MAE-DB and Portland cement. PLoS ONE.

[27] Lone S.B., Zeeshan R., Khadim H., Khan M.A., Khan A.S., Asif A. (2024). Synthesis, monomer conversion, and mechanical properties of polylysine based dental composites. J. Mech. Behav. Biomed. Mater..

[28] Alania Y., Natale L.C., Nesadal D., Vilela H., Magalhães A.C., Braga R.R. (2019). In vitro remineralization of artificial enamel caries with resin composites containing calcium phosphate particles. J. Biomed. Mater. Res. B Appl. Biomater..

[29] Weir M.D., Ruan J., Zhang N., Chow L.C., Zhang K., Chang X., Bai Y., Xu H.H.K. (2017). Effect of calcium phosphate nanocomposite on in vitro remineralization of human dentin lesions. Dent. Mater..

[30] Mohn N., Par M., Gubler A., Tauböck T.T. (2024). Marginal integrity of prototype bioactive glass-doped resin composites in class II cavities. Clin. Oral Investig..

[31] Xie X.J., Xing D., Wang L., Zhou H., Weir M.D., Bai Y.-X., Xu H.H. (2017). Novel rechargeable calcium phosphate nanoparticle-containing orthodontic cement. Int. J. Oral Sci..

[32] Tezvergil-Mutluay A., Seseogullari-Dirihan R., Feitosa V., Cama G., Brauer D., Sauro S. (2017). Effects of composites containing bioactive glasses on demineralized dentin. J. Dent. Res..

[33] Rahmanpanah S., Seifi M., Gharavi Z., Sadighnia N., Amdjadi P. (2023). Evaluation of shear bond strength and enamel remineralizing effect of experimental orthodontic composite containing nano-hydroxyapatite: An in vitro study. Int. Orthod..

[34] Weir M.D., Chow L.C., Xu H.H.K. (2012). Remineralization of demineralized enamel via calcium phosphate nanocomposite. J. Dent. Res..

[35] Park K.J., Schneider H., Haak R. (2015). Assessment of defects at tooth/self-adhering flowable composite interface using swept-source optical coherence tomography (SS-OCT). Dent. Mater..

[36] Alb C., Alb S., Dudea D., Nicola C., Moldovan M., Culic B., Buduru S., Mesaros A. (2010). In vitro testing of an experimental dental composite resin and adhesive system. Rev. Med. Chir. Soc. Med. Nat. Iasi.

[37] Brueckner C., Schneider H., Haak R. (2017). Shear bond strength and tooth-composite interaction with self-adhering flowable composites. Oper. Dent..

[38] Hu C., Lin Y., Yang Y., Wang L., Liang H., Wu J., He G., Shao L. (2022). High-performance dental composites based on hierarchical reinforcements. J. Dent. Res..

[39] Khan A.S., Hassan K.R., Bukhari S.F., Wong F.S.L., Rehman I.U. (2012). Structural and in vitro adhesion analysis of a novel covalently coupled bioactive composite. J. Biomed. Mater. Res. B Appl. Biomater..

[40] Nikpour S., Saffar Shahroudi A., Saffarpour A., Akhavan A., Sodagar A. (2021). Shear bond strength of self-adhesive composite containing TiO_2_ and SiO_2_ nanoparticles with an additional etching step for orthodontic brackets bonding to enamel. Folia Medica.

[41] Sterzenbach G., Karajouli G., Tunjan R., Spintig T., Bitter K., Naumann M. (2015). Damage of lithium-disilicate all-ceramic restorations by an experimental self-adhesive resin cement used as core build-ups. Clin. Oral Investig..

[42] Goracci C., Margvelashvili M., Giovannetti A., Vichi A., Ferrari M. (2013). Shear bond strength of orthodontic brackets bonded with a new self-adhering flowable resin composite. Clin. Oral Investig..

[43] Czasch P., Ilie N. (2013). In vitro comparison of mechanical properties and degree of cure of a self-adhesive and four novel flowable composites. J. Adhes. Dent..

[44] Noushad M., Zulkifli N.S.C., Ab Rahman I., Husein A., Mohamad D., Ismail A.R. (2013). Nanosilica from rice husk as fillers in dental nanocomposites—A preliminary study. Proceedings of the 2013 International Conference on Advanced Nanomaterials and Emerging Engineering Technologies (ICANMEET 2013).

[45] Yusoff N.M., Johari Y., Ab Rahman I., Mohamad D., Khamis M.F., Husein A., Ariffin Z. (2018). Hardness of flowable resin composite from rice husk. J. Mech. Eng..

[46] Yusoff N.M., Johari Y., Ab Rahman I., Mohamad D., Khamis M.F., Ariffin Z., Husein A. (2019). Physical and mechanical properties of flowable composite incorporated with nanohybrid silica synthesised from rice husk. J. Mater. Res. Technol..

[47] Noushad M., Ab Rahman I., Husein A., Mohamad D. (2016). Nanohybrid dental composite using silica from biomass waste. Powder Technol..

[48] Ismail N.H., You L.S., Abd Jalil A.N., Ab Ghani N.B., Awang R.A. (2020). Physical and mechanical properties analyses of zirconia reinforced experimental nanohybrid dental composite (NHDC) from rice husk. Malays. J. Microsc..

[49] Raja K.K., Hari P., Chin M.Q.K., Singbal K., Fareez I.M. (2019). Color stability of a new rice husk composite in comparison with conventional composites after exposure to commonly consumed beverages in Malaysia. Int. J. Dent..

[50] Lin G.S.S., Ghani N., Ismail N., Singbal K., Murugeshappa D., Mamat N. (2020). Fracture strength of endodontically treated lateral incisors restored with new zirconia reinforced rice husk nanohybrid composite. J. Clin. Exp. Dent..

[51] Lin G.S.S., Nik Abdul Ghani N.R., Ismail N.H., Singbal K.P., Yusuff N.M.M. (2020). Polymerization shrinkage and degree of conversion of new zirconia-reinforced rice husk nanohybrid composite. Eur. J. Dent..

[52] Bhat I.K., Patnaik A., Kumar S.R. (2017). Investigation of wear characteristics of dental composite reinforced with rice husk-derived nanosilica filler particles. Handbook of Composites from Renewable Materials.

[53] Al-Rawas M., Johari Y., Mohamad D., Khamis M.F., Ahmad W.M.A.W., Ariffin Z., Husein A. (2021). Water sorption, solubility, degree of conversion, and surface hardness and topography of flowable composite utilizing nano silica from rice husk. J. Mater. Res. Technol..

[54] Azlisham N.A.F., Johari Y., Mohamad D., Yhaya M.F., Mahmood Z. (2025). Physicomechanical properties and polymerization shrinkage of the newly developed radiopaque flowable composite derived from rice husk. Polym. Compos..

[55] Azlisham N.A.F., Johari Y., Mohamad D., Yhaya M.F., Mahmood Z. (2023). Degree of conversion and physicomechanical properties of newly developed flowable composite derived from rice husk using urethane dimethacrylate monomer. Proc. Inst. Mech. Eng. Part H J. Eng. Med..

[56] Odermatt R., Par M., Mohn D., Wiedemeier D.B., Attin M., Tauböck T.T. (2020). Bioactivity and physico-chemical properties of dental composites functionalized with nano-vs. micro-sized bioactive glass. J. Clin. Med..

[57] Travan A., Donati I., Marsich E., Bellomo F., Achanta S., Toppazzini M., Semeraro S., Scarpa T., Spreafico V., Paoletti S. (2010). Surface modification and polysaccharide deposition on BisGMA/TEGDMA thermoset. Biomacromolecules.

[58] Abdulmajeed A.A., Kokkari A.K., Käpylä J., Massera J., Hupa L., Vallittu P.K., Närhi T.O. (2014). In vitro blood and fibroblast responses to BisGMA-TEGDMA/bioactive glass composite implants. J. Mater. Sci. Mater. Med..

[59] Păstrav M., Păstrav O., Chisnoiu A.M., Chisnoiu R.M., Cuc S., Petean I., Saroși C., Feștilă D. (2024). Properties of nanohybrid dental composites—A comparative in vitro study. Biomedicines.

[60] Thorat S.B., Diaspro A., Salerno M. (2014). In vitro investigation of coupling-agent-free dental restorative composite based on nano-porous alumina fillers. J. Dent..

[61] Wang R., Zhang M., Liu F., Bao S., Wu T., Jiang X., Zhang Q., Zhu M. (2015). Investigation on the physical-mechanical properties of dental resin composites reinforced with novel bimodal silica nanostructures. Mater. Sci. Eng. C.

[62] Sivakumar J.S.K., Prasad A.S., Soundappan S., Ragavendran N., Ajay R., Santham K. (2016). A comparative evaluation of microleakage of restorations using silorane-based dental composite and methacrylate-based dental composites in Class II cavities: An in vitro study. J. Pharm. Bioallied Sci..

[63] Lotfi L., Javadpour J., Naimi-Jamal M.R. (2018). Biological and nano-indentation properties of polybenzoxazine-based composites reinforced with zirconia particles as a novel biomaterial. Bio-Med. Mater. Eng..

[64] Jassé F.F., de Campos E.A., Lefever D., Di Bella E., Salomon J.P., Krejci I., Ardu S. (2013). Influence of filler charge on gloss of composite materials before and after in vitro toothbrushing. J. Dent..

[65] Braun A., Glockmann A., Krause F. (2013). Spectrophotometric evaluation of a novel aesthetic composite resin with respect to different backgrounds in vitro. Odontology.

[66] Askar H., Schwendicke F., Lausch J., Meyer-Lueckel H., Paris S. (2018). Modified resin infiltration of non-, micro- and cavitated proximal caries lesions in vitro. J. Dent..

[67] Paris S., Meyer-Lueckel H. (2010). Infiltrants inhibit progression of natural caries lesions in vitro. J. Dent. Res..

[68] Roman A., Páll E., Moldovan M., Rusu D., Şoriţău O., Feştilă D., Lupşe M. (2016). Cytotoxicity of experimental resin composites on mesenchymal stem cells isolated from two oral sources. Microsc. Microanal..

[69] Chadda H., Naveen S.V., Mohan S., Satapathy B.K., Ray A.R., Kamarul T. (2016). Cytotoxic evaluation of hydroxyapatite-filled and silica/hydroxyapatite-filled acrylate-based restorative composite resins: An in vitro study. J. Prosthet. Dent..

[70] De Oliveira D.C.R.S., Silva C.B., Muniz B.V., Volpato M.C., Costa A.R., Sinhoreti M.A.C. (2014). Effect of 4-(N,N-dimethylamino) phenethyl alcohol on degree of conversion and cytotoxicity of photo-polymerized CQ-based resin composites. Braz. Dent. J..

[71] Rozza B.Y., El-Refai D.A., Essawy H.A., Alian G.A. (2024). Effect of silanization of poly(urea-formaldehyde) microcapsules on the flexural strength and self-healing efficiency of an experimental self-healing dental resin composite: An in vitro study. J. Mech. Behav. Biomed. Mater..

[72] (2019). Dentistry—Polymer-Based Restorative Materials.

[73] (2009). Biological Evaluation of Medical Devices—Part 5: Tests for In Vitro Cytotoxicity.

[74] (2013). Dentistry—Adhesion—Notched-Edge Shear Bond Strength Test.

[75] (2023). Dentistry—Test Methods for Tensile Bond Strength to Tooth Structure.

[76] Mohammed S.F., Yhaya M.F., Nongman A.F., Al-Rawas M., Arbilei M.N., Noorani T.Y. (2025). From Industry to Dentistry: A Comprehensive Review of Zeolite as a Next-Generation Multifunctional Filler for Enhanced Mechanical Reinforcement and Antimicrobial Efficacy. Dent. J..

