# Supplementary material for: Insights on Novel Dental Resin Composites: A Scoping Review
Source: Polymers (Basel). 2026 Oct 14;18(20):2516. doi: 10.3390/polym18202516 (PMC13709035; doi:10.3390/polym18202516)
Supplement: Supplementary file 1 [file polymers-18-02516-s001.zip › polymers-4591230-supplementary.pdf]

Table S1. Category-level methodological appraisal.

Category and Study Range	Methodological Strengths	Main Limitations	Implication for Interpretation
Antibacterial strategies [13–26]	Matched controls help isolate additive effects [13,14]. Some studies combine mechanical and cell testing [21,24] or evaluate activity after aging [15,26]. Reported replication includes six specimens per group and three independent repeats for compressive testing, with ISO 4049 cited for specimen preparation [13].	Models range from single-species assays to defined mixtures and saliva-derived microcosms [13,14,16]. Cell testing and mechanical assessment are not consistently combined; precursor characterization alone does not demonstrate antibacterial efficacy [19]. A sample-size justification was not reported in [13].	Interpret activity by formulation, model and exposure. Short-term inhibition cannot establish sustained clinical caries prevention or biological safety.
Remineralizing strategies [27–34]	Lesion microradiography directly evaluates mineral change [28,29,34]. Selected studies combine aging with bond or flexural testing [29,31,33]. A power-based calculation supported ten teeth per group, and ISO/TS 11405:2003 was cited for storage and thermocycling [33].	Solution precipitation and ion release are less direct than tooth-lesion measurements. Multiple beams may originate from the same tooth [32]; biological and mechanical outcomes are not always tested together.	Separate mineralization surrogates from tooth remineralization and clinical benefit. Assess functional gains alongside measured mechanical tradeoffs.
Bonding and interfaces [35–43]	Tooth-substrate comparisons, thermocycling and longitudinal interface/crack observations address bonding durability [35,37,40–42]. Shear-bond testing reported ten teeth per material–substrate–aging condition and referenced ISO/TS 11405, with a modified thermocycling regimen [37].	Substrate, adhesive strategy, and aging differ between studies. Additional enamel etching can limit claims of self-adhesion [40]. Different adhesives complicate resin-only comparisons [36]; some test geometries are modified [43].	Keep enamel and dentin, immediate and aged outcomes, and adhesive systems distinct. Similar laboratory bond values do not establish equivalent clinical retention.
Rice-husk-derived materials [44–55]	Several studies use commercial comparators and named test methods [47,53–55]. Selected experiments include beverage exposure or thermocycling [49,50]. Depth-of-cure testing reported seven specimens	A sample-size justification was not reported in [54]. Initial-property testing in [47,54,55] provides limited evidence of long-term durability; extended mechanical aging and biological testing were not reported in these studies.	Commercial comparisons may differ in several formulation components; initial properties do not demonstrate long-term durability or sustainability.

	per group and referenced ISO 4049 [54].		
Other innovations [56–71]	Selected studies combine surface biology with mechanics [57], use prolonged chemical challenge [67], or test cells from oral tissues [68]. Mechanical testing reported ten specimens per group; fracture-toughness specimen preparation referenced ASTM E399-90 [71].	Targets and methods are highly heterogeneous. Model-based accelerated aging [60], short cell exposures [69,70] and a single healing protocol [71] address different questions. Independent replication is unclear in some reports [63,64,69].	Evaluate each technology against its intended endpoint. Cell viability, gloss, infiltration and crack recovery are not interchangeable measures of overall performance.

Footnote: This table summarizes methodological features and limitations by category, with selected study references supporting each observation. Findings do not necessarily apply to every study within a category. The appraisal is descriptive and does not provide individual-study risk-of-bias ratings or an overall quality score.
